# Synchronous pituitary and pineal gland lesions presenting with panhypopituitarism in a patient with widespread colorectal cancer: a case report

**DOI:** 10.1093/omcr/omab030

**Published:** 2021-05-24

**Authors:** Tristan Struja, Joël Capraro

**Affiliations:** Department of Endocrinology and Diabetology, Medical University Clinic, Kantonsspital Aarau, Aarau, Switzerland

## Abstract

A 60-year-old man presented to our hospital because of febrile neutropenia due to chemotherapy for his metastasized colon cancer. During hospital stay, polyuria and polydipsia were noted. He reported that his thirst had become increasingly intolerable over the last weeks. Diabetes mellitus was diagnosed. Polyuria and polydipsia persisted despite euglycemia under insulin treatment. Because of thirst and high urine volumes during the night, diabetes insipidus was suspected. Endocrinological work up revealed complete panhypopituitarism with impairment of all anterior and posterior axes. After substitution with hydrocortisone, levothyroxine and desmopressin symptoms resolved. MRI of the brain revealed one round, contrast enhancing lesion in the pituitary stalk and one in the pineal gland. Because of extensive extracerebral metastasis and poor performance status, the patient opted for radiation therapy only. He died 2.5 months later due to non-cerebral manifestations of his cancer before cerebral radiotherapy could be initiated.

## INTRODUCTION

While cancer metastasis to the pituitary is a very rare event, metastases to the pituitary stalk or the pineal gland are even scarcer. Astonishingly, the reported cases usually do not present with extensive hormonal dysfunctions. Usually, the reported patients suffered from breast or lung cancer [1]. Here, we present the case of a man who suffered from colon cancer with extensive metastases. During his illness, he potentially developed a metastasis to the pineal gland and another to the pituitary stalk.

## CASE REPORT

A 60-year-old man was treated at our hospital because of his metastasized colon cancer diagnosed one and a half year prior. Primary site was the ascending colon. After the initial diagnosis and hemicolectomy, an adjuvant chemotherapy with oxaliplatin and capecitabine (XELOX) followed over 3 months. Besides his cancer, he was being treated for hypertension, dyslipidaemia, sleep apnoea, primary hypothyroidism and asthma.

**Figure 1:**
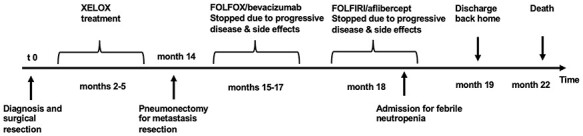
Timeline depicting the pertinent events in the course of the disease. Caution: Drawing not to scale. To protect the confidentiality of the patient, real dates were substituted with months after diagnosis. Abbreviations: XELOX: oxaliplatin and capecitabine. FOLFOX: folinic acid, fluorouracil, oxaliplatin. FOLFIRI: folinic acid, fluorouracil, irinotecan.

One year later, lung metastases were noted (see Fig. 1 for timeline of events), and a left-sided pneumectomy was performed. Additionally, a second line therapy with folinic acid, fluorouracil, oxaliplatin (FOLFOX) and bevacizumab was initiated. The tumour was found to have mutations in PIK3CA (exon 21, c.3140AQ > G; p.(His1047Arg)) and KRAS (exon 4, c.436G > A; p.(Ala146Thr)). There was no microsatellite instability, HER 2 was negative, and NRAS and BRAF were wildtype. Because of progressive disease, treatment was switched to a third line with folinic acid, fluorouracil, irinotecan (FOLFIRI) and aflibercept. Consecutively, he presented to our hospital because of febrile neutropenia. During hospital stay, polyuria and polydipsia were noted. He reported that his thirst had become increasingly intolerable over the last weeks. Capillary blood glucose values up to 11.2 mmol/l (180 mg/dl) and a haemoglobin A1c of 6.9% were detected and diabetes mellitus was diagnosed. Polyuria and polydipsia persisted despite euglycemia under insulin treatment. Fluid intake reached up to 6.3 l per day. Urine volume was as high as 8.4 l per day. Especially because of persistent thirst and high urine volumes during the night, diabetes insipidus (DI) was suspected. Endocrinological work up revealed complete panhypopituitarism with impairment of all anterior and posterior axes (see Table 1). We did not perform any endocrinological function test as the hormonal deficiencies were that extensive. While a random copeptin was non-diagnostic, hypertonic saline or water deprivation testing was deemed unnecessary because of the clinical picture and patient comfort. After substitution with hydrocortisone, desmopressin, and increase of his levothyroxine dose symptoms resolved. Most strikingly, water intake and urine volume dropped to 2–3 l per day.

**Table 1:** Laboratory values of endocrinological work up

Parameter	(norm)	Values	Remarks
HbA1c	(<6.5%)	6.9%	
Glucose venous	(<5.5 mmol/l)	12.6 mmol/l	
TSH	(0.4–4.0 mU/l)	0.1 mU/l	
Free T4	(10.0–22.7 pmol/l)	8.0 pmol/l	
Copeptin	(1.2–16.4 pmol/l)	3.6 pmol/l	Measured at a sodium of 143 mmol/l
Cortisol baseline	(>500 nmol/l)	<35.0 nmol/l	
IGF-1	(7.7–25.5 nmol/l)	11.4 nmol/l	
Testosterone	(5–32 nmol/l)	<0.15 nmol/l	
LH	(1.0–8.6 U/l)	<0.2 U/l	
FSH	(0.7–11 U/l)	2.6 U/l	
Prolactin	(2.7–17 μg/l)	34.5 μg/l	

Legend: HbA1c, haemoglobin A1c; TSH, thyrotropin; free T4, free thyroxine; Copeptin, peptide derived from the C-terminus of the pre-pro-hormone complex of vasopressin, neurophysin II, and copeptin, equimolar secretion with vasopressin; IGF-1, insulin like growth factor 1; LH, luteinizing hormone; FSH, follicle-stimulating hormone

**Figure 2:**
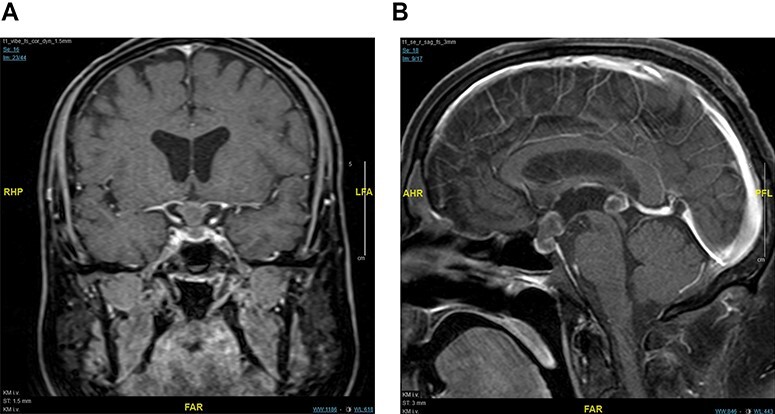
Image A, coronal MRI with intravenous contrast depicting a pituitary stalk metastasis (white arrow). Image B, sagittal MRI with intravenous contrast depicting metastases to the pituitary stalk (white arrow) and pineal gland (white arrowhead).

MRI of the brain revealed one round, contrast enhancing lesion in the pituitary stalk (see Fig. 2, image A and B, white arrow) and one in the pineal gland (Image B, white arrowhead). Otherwise, there were no further intracranial lesions noted. Ophthalmological assessment did not demonstrate any visual field deficits or vertical gaze palsy. Because of extensive disease progression and poor performance status, the patient opted for radiation therapy only, and we did not obtain tissue samples from the lesions. Because of the rapidly progressive disease, he died 2.5 months later at home (cause of death unknown) before the radiation therapy could take place.

## DISCUSSION

Usually, simultaneous mass lesions in the suprasellar region and the pineal gland along with symptoms of panhypopituitarism are the typical presentation of an intracranial germ cell tumour, especially germinoma [2]. Because the colon cancer in our case was growing very aggressively, showed extensive extracranial metastases, and our patient was already 60 years old, we think that intracranial metastases are a valid differential diagnosis. Characteristically, intracranial germ cell tumours present in almost 90% of the cases before the age of 20 and show increased serum and cerebrospinal fluid levels of alpha-fetoprotein and β-human chorionic gonadotropin [2]. As we did not test for these markers and have not sampled tissue from these lesions, we cannot rule out the possibility of an unusual late presentation.

Metastasis to the pituitary is a very rare event. Up to 2018, only 289 cases were reported in the literature [1]. Most of the cases were due to breast or lung cancer. While metastasis to the pituitary stalk is even scarcer, patients usually do not present with such extensive hormonal dysfunctions [3].

Due to the scarcity of pituitary metastases, robust data on the efficacy of radiation therapy are missing. Data from pituitary adenoma radiation show a tumour volume control rate ranging from 50% up to 97% [4]. However, many previous studies where heterogeneous and radiation techniques changed substantially in the last years (i.e. transition from fractionated radiotherapy to stereotactic focused radiation). In pituitary adenoma, progression-free survival was reported to be 25% lower compared to an untreated group after 10 years of follow-up.

Interestingly, this presentation also included a metastasis to the pineal gland. Pineal gland metastases account for only 0.4 to 3.8% of all intracranial metastases and usually are caused by lung cancer [5]. While this lesion was not clinically relevant in our case, loss of melatonin production can lead to sleep disturbances. Obstruction of cerebrospinal fluid flow can lead to hydrocephalus [6].

Originally, a water deprivation test over 8 to 12 h was used to diagnose DI, often giving equivocal results. Vasopressin measurements are hampered by its low half-life and assay difficulties [7]. Herein, copeptin is a valuable alternative. It is equimolarly secreted with vasopressin, has a much longer half-life and can be measured with commercially available immunoassays. Instead of a water deprivation test, we now use a 3% saline infusion test over 3h in ambulatory patients. A sodium level ≥150 mmol/l and a copeptin ≤4.9 pmol/l are diagnostic for central DI [8].

We described the case of a 60-year-old man with diabetes mellitus, panhypopituitarism and pituitary stalk and pineal mass lesions. These lesions have a wide differential diagnosis and can either have been caused by a germinal cell tumour or metastases. The older age of our patient and his extensive colon cancer makes metastatic colorectal cancer a valid differential for the aetiology of the presented case. Also, it is important to stay vigilant for alternative diagnoses such as DI if sympotms do not seolve after initial treatment of diabetes mellitus.

## References

[1] Javanbakht A , D'Apuzzo M, Badie B, Salehian B. Pituitary metastasis: a rare condition. Endocr Connect 2018;7:1049–57. 10.1530/EC-18-0338 PMC6198191 30139817

[2] Jorsal T , Rorth M. Intracranial germ cell tumours. A review with special reference to endocrine manifestations. Acta Oncol 2012;51:3–9. 22150165 10.3109/0284186X.2011.586000

[3] Turcu AF , Erickson BJ, Lin E, Guadalix S, Schwartz K, Scheithauer BW, et al. Pituitary stalk lesions: the Mayo Clinic experience. J Clin Endocrinol Metab 2013;98:1812–8. 23533231 10.1210/jc.2012-4171

[4] Castinetti F . Radiation techniques in aggressive pituitary tumours and carcinomas. Rev Endocr Metab Disord 2020;21:287–92. 32064542 10.1007/s11154-020-09543-y

[5] Taydas O , Yesilyurt M, Ogul Y, Ogul H. Isolated pineal gland metastasis of acute lymphocytic leukemia: case report. Cancer Biol Ther 2020;21:503–5. 32208886 10.1080/15384047.2020.1735605 PMC7515532

[6] Cipolla-Neto J , Amaral FGD. Melatonin as a hormone: new physiological and clinical insights. Endocr Rev 2018;39:990–1028. 30215696 10.1210/er.2018-00084

[7] Christ-Crain M , Fenske W. Copeptin in the diagnosis of vasopressin-dependent disorders of fluid homeostasis. Nat Rev Endocrinol 2016;12:168–76. 26794439 10.1038/nrendo.2015.224

[8] Fenske W , Refardt J, Chifu I, Schnyder I, Winzeler B, Drummond J, et al. A Copeptin-based approach in the diagnosis of diabetes insipidus. N Engl J Med 2018;379:428–39. 30067922 10.1056/NEJMoa1803760

